# Adverse Reactions Following Intradermal Injection of Exosome‐Based Formulations: A Case Series

**DOI:** 10.1111/jocd.70520

**Published:** 2025-10-15

**Authors:** Kui Young Park

**Affiliations:** ^1^ Department of Dermatology Chung‐Ang University College of Medicine Seoul South Korea

**Keywords:** adverse reaction, cosmetic injection, exosomes, granuloma

## Abstract

**Background:**

Exosome‐containing formulations have recently gained popularity in aesthetic dermatology because of their regenerative and anti‐inflammatory properties. Although approved for topical use, intradermal injection of these cosmetic products remains off‐label and unregulated in many countries, including South Korea.

**Aims:**

To describe four cases of severe cutaneous adverse reactions following the intradermal injection of unregulated exosome‐based formulations.

**Methods:**

Four adult women developed persistent erythema, nodules, granulomatous inflammation, and scarring following intradermal administration of exosome‐containing products in a nonclinical setting. The clinical course, treatment, and outcomes of the patients were documented.

**Results:**

All the patients experienced persistent inflammatory lesions. Treatments included corticosteroids (systemic and intralesional), laser therapy, and surgical removal. The outcomes varied, with all patients experiencing incomplete resolution and residual scarring.

**Conclusion:**

Intradermal injection of unapproved exosome‐based formulations may cause serious and persistent cutaneous complications. This case series underscores the need for regulatory enforcement, practitioner education, and public awareness to prevent off‐label use and protect patient safety in aesthetic practice.

## 1 Introduction

Exosomes, nanosized extracellular vesicles derived from stem cells, have recently attracted significant interest in dermatology because of their regenerative and anti‐inflammatory properties [1, 2]. Exosomes modulate immune responses and promote tissue repair by mediating cell‐to‐cell communication, making them appealing for applications in wound healing, skin rejuvenation, and hair regeneration [3, 4]. However, to date, exosome‐based products have been approved and marketed primarily as topical cosmetics without formal regulatory approval for injectable use in most countries, including South Korea.

Despite this, intradermal injection of exosome‐based formulations has emerged as an off‐label practice in aesthetic medicine, with proponents claiming greater efficacy than the topical application [5]. The safety of this use remains poorly defined, and recent reports have raised concerns regarding its immunogenicity, contamination, and long‐term cutaneous or systemic complications [6, 7]. Here, we describe four cases of adverse cutaneous reactions following the intradermal injection of exosome‐based formulations, underscoring the urgent need for regulatory oversight, practitioner education, and greater awareness of the risks associated with unauthorized exosome injections.

## 2 Case Reports

This case series describes four patients who developed severe cutaneous inflammatory reactions following the intradermal injection of a cosmetic exosome‐containing formulation. All procedures were performed outside of medical supervision, in non‐dermatology clinics or beauty salons. The patients subsequently sought treatment at our dermatology clinic. Written informed consent was obtained from all patients for the publication of clinical information and photographs. In accordance with our institutional review board policy, ethical approval was not required for this retrospective case series because it involved noninterventional data collection and anonymized clinical observations.

### 2.1 Case 1

**FIGURE 1:**
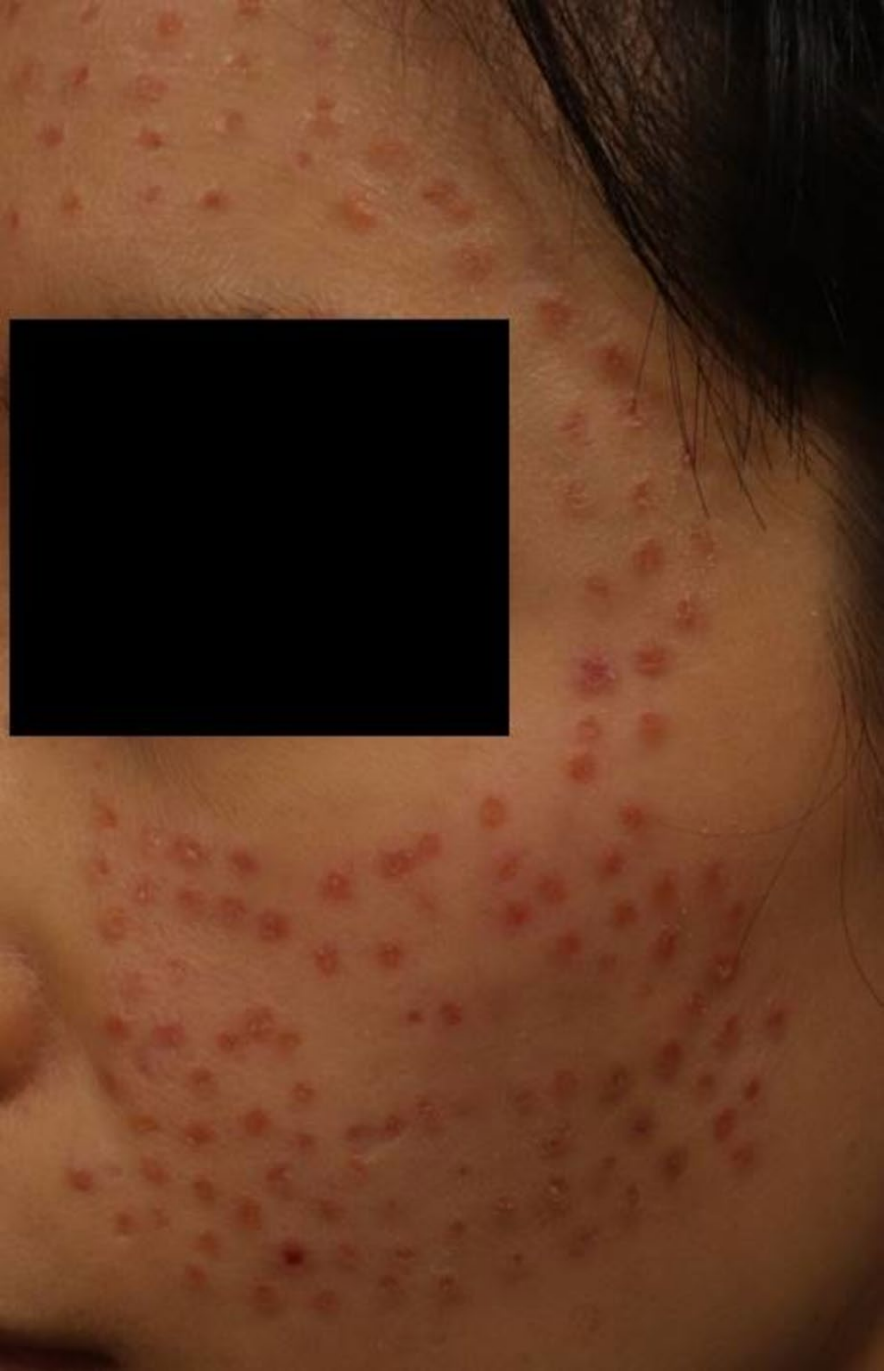
Multiple erythematous papules and nodules on the cheek of a 40‐year‐old woman following intradermal injection of an exosome‐containing formulation at a non‐dermatologist clinic. The lesions were distributed in a punctate pattern at the injection sites and persisted despite treatment with corticosteroids and laser therapy.

A 40‐year‐old woman received intradermal injections of an exosome‐containing formulation at a non‐dermatological clinic. Within weeks, the patient developed persistent erythema and nodules at the injection site. Despite multiple interventions, including corticosteroid therapy and laser treatment, the symptoms persisted (Figure 1).

### 2.2 Case 2

**FIGURE 2:**
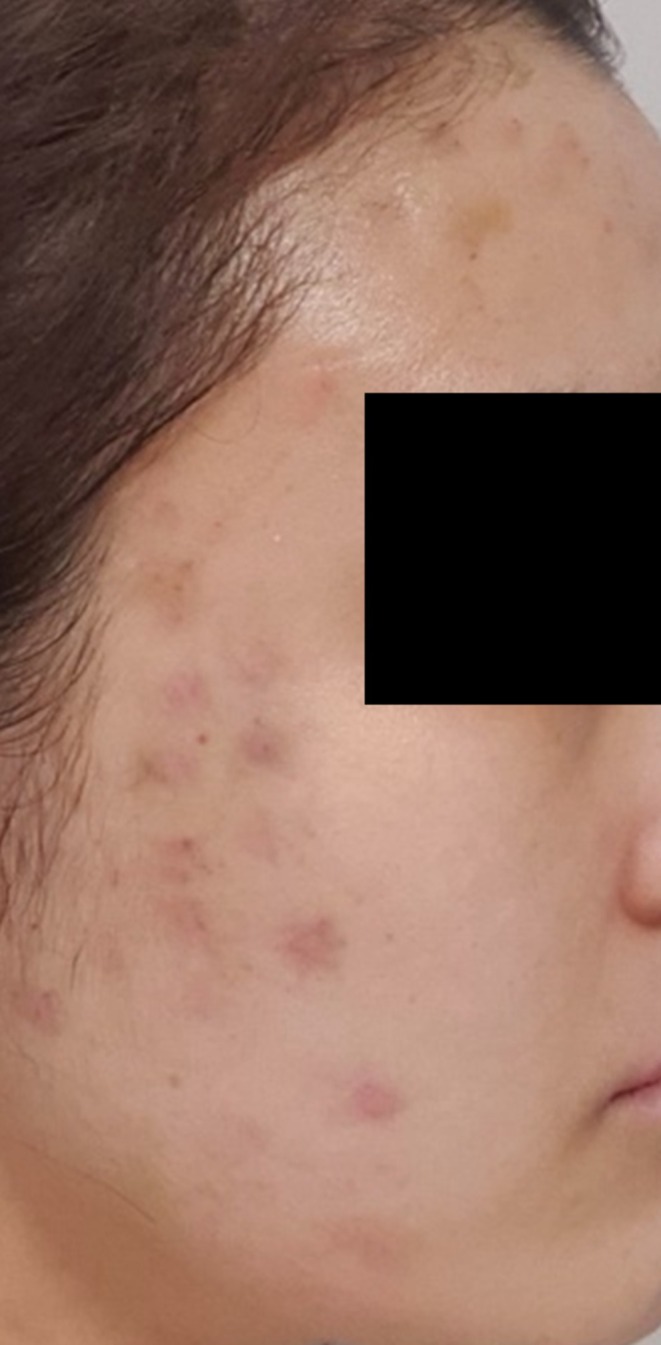
Post‐inflammatory erythematous macules and residual indurated nodules on the cheek of a 31‐year‐old woman, observed several months after intradermal injection of an exosome‐containing formulation. Initial erythema and swelling partially subsided with corticosteroid therapy and laser treatments, but residual erythema, pigmentation, and subcutaneous nodules persisted.

A 31‐year‐old woman received an intradermal injection of an unspecified exosome‐containing formulation at a non‐dermatological clinic in February 2021. The patient experienced immediate erythema, swelling, and warmth at the injection site. Treatments, including Local Dynamic Micro‐massage ultrasound therapy (LDM, Wellcomet GmbH, Germany), 532 and 1064 nm dual‐wavelength laser (Excel V, Cutera Inc., USA), and chemical peeling, were performed but failed to fully resolve the lesions (Figure 2). Intralesional corticosteroids resulted in atrophic scarring, and the patient eventually discontinued medical treatment and resorted to self‐care.

### 2.3 Case 3

**FIGURE 3:**
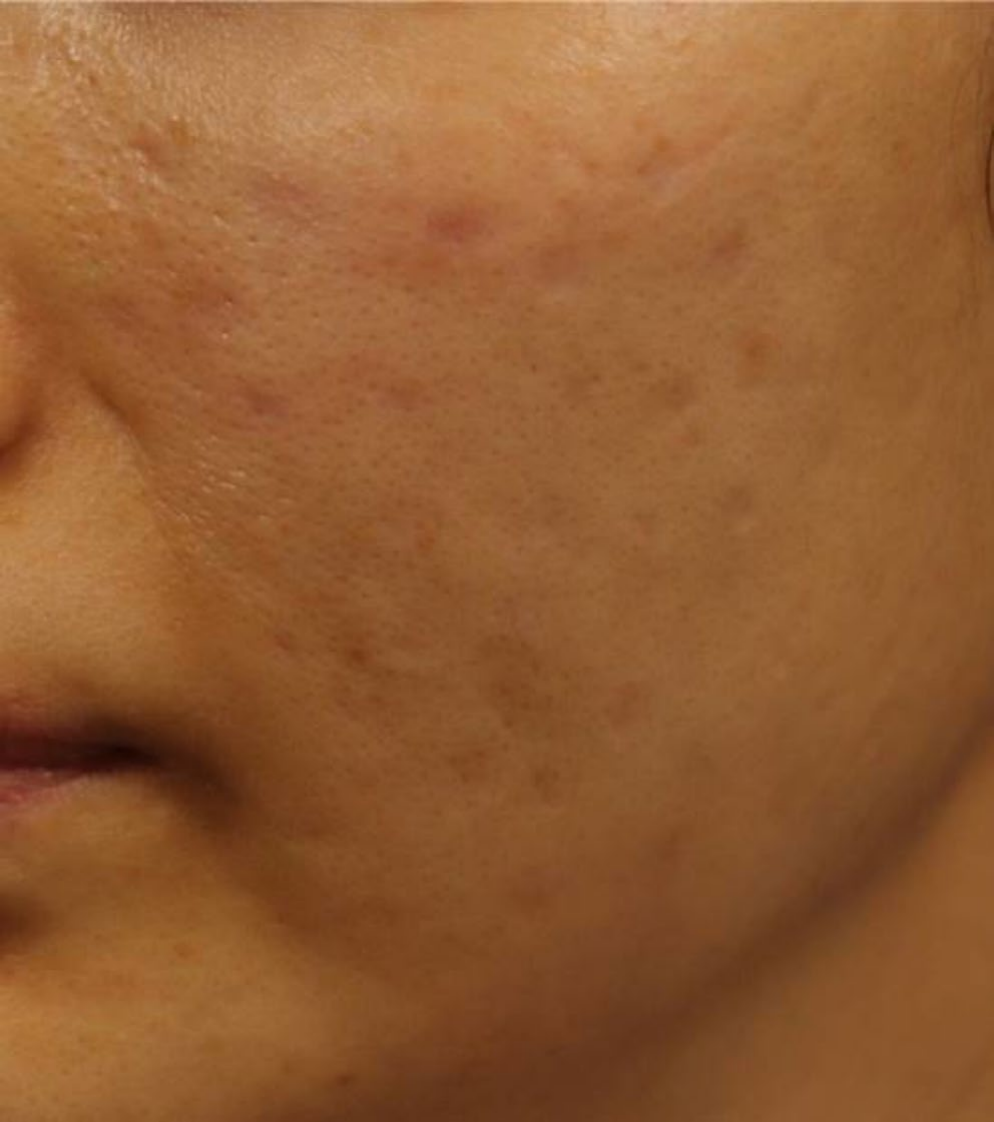
Multiple post‐inflammatory hyperpigmented macules and shallow atrophic scars on the cheek of a 42‐year‐old woman following intradermal injection of an exosome‐containing formulation. Despite repeated interventions including corticosteroid therapy, incision and drainage, and laser treatment, the patient exhibited long‐standing textural changes and pigmentation.

A 42‐year‐old woman received intradermal injections of an unspecified exosome‐containing formulation in November 2018 at a non‐dermatological clinic. She developed multiple nodular skin lesions that were subsequently treated with incision and drainage, intralesional triamcinolone, systemic corticosteroids, and topical steroids. Despite these interventions, persistent scarring required multiple laser treatments (Figure 3).

### 2.4 Case 4

**FIGURE 4:**
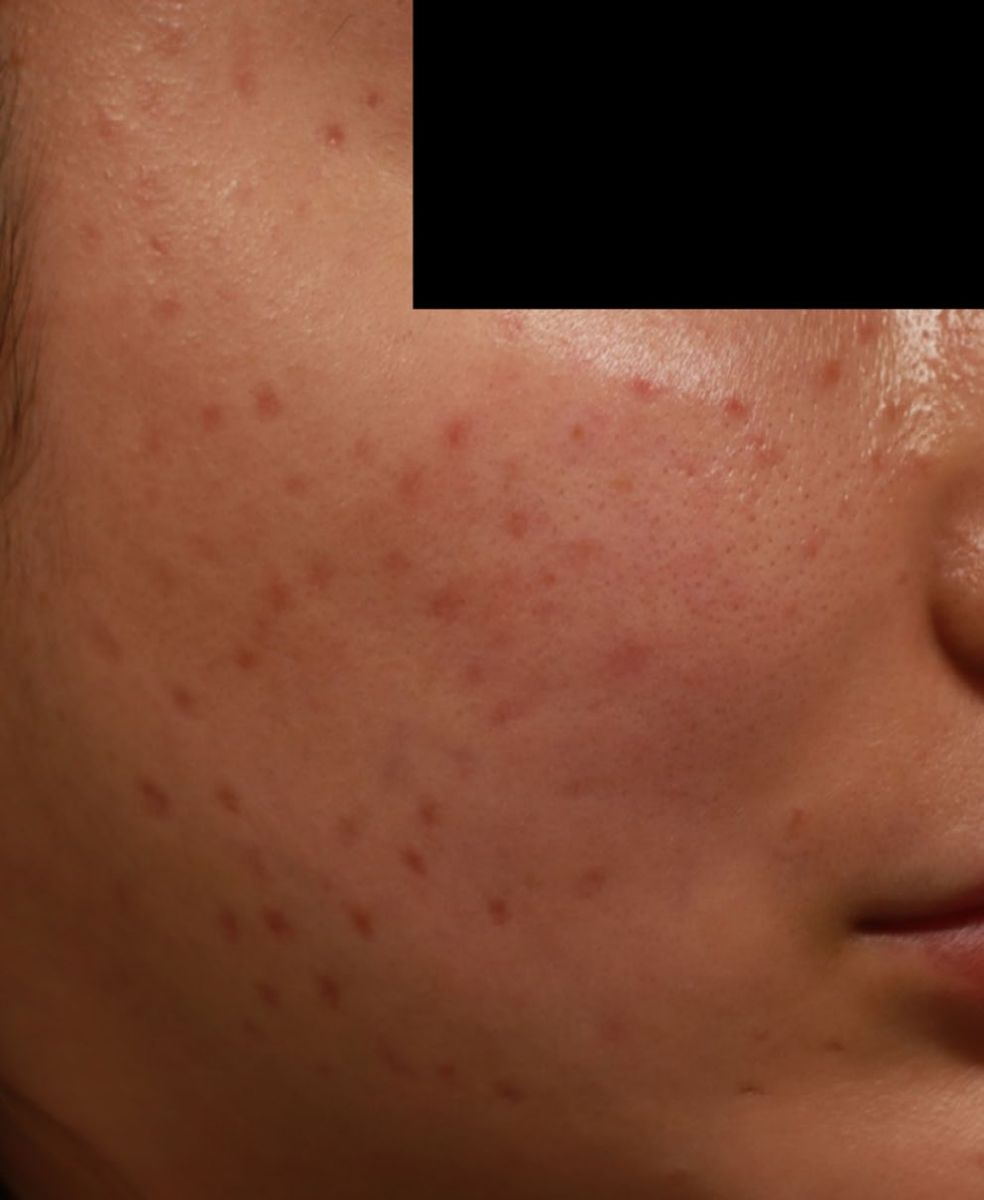
Persistent erythematous macules and scattered inflammatory papules on the cheek of a 33‐year‐old woman following intradermal injection of an exosome‐containing formulation at a beauty salon. Despite undergoing multiple cannula‐based removals, corticosteroid injections, platelet‐rich plasma therapy, and laser treatments, the patient exhibited residual erythema and papular lesions.

A 33‐year‐old woman received injections of an unidentified exosome‐containing formulation at a beauty salon in January 2020. Severe inflammatory reactions led to multiple removal procedures using an 18G cannula, repeated corticosteroid injections, and platelet‐rich plasma (PRP) therapy. Despite extensive laser treatment, residual scarring persisted (Figure 4).

## 3 Discussion

Off‐label intradermal injection of exosome‐containing formulations poses significant risks, primarily due to the potential for unintended immune and inflammatory responses. Unlike pharmaceuticals or medical‐grade injectables, which undergo rigorous safety evaluations, cosmetic products are generally formulated for topical use, where the stratum corneum acts as a barrier to prevent systemic absorption. When introduced directly into the dermis, various excipients, preservatives, and biologically active compounds can trigger unexpected adverse effects. Delayed‐type hypersensitivity reactions, granulomatous inflammation, and persistent nodular lesions have been reported following cosmetic injections. These reactions can be attributed to immune responses against exogenous proteins, plant‐derived extracts, or stabilizers commonly found in cosmetic formulations [5, 6, 7].

Granuloma formation is a particularly concerning adverse effect, often arising from a chronic foreign body reaction or a delayed immune response to injected substances. Histological evaluations typically reveal epithelioid histiocytes and multinucleated giant cells surrounding the injected material, consistent with a granulomatous reaction. These findings suggest that, unlike medical‐grade injectables, cosmetic ampoules may contain components that are not fully metabolized or safely cleared by the body, leading to persistent inflammation. The presence of botanical extracts, polysaccharides, and synthetic stabilizers may further exacerbate these immune‐mediated reactions, particularly in individuals with pre‐existing sensitivities or compromised immune tolerance. Notably, Tawanwongsri et al. described a case of skin necrosis following the intradermal injection of a lyophilized exosome formulation, with histopathological evidence of both granulomatous inflammation and necrotic vascular changes [5].

In support of our findings, several recently published case reports described comparable adverse events. Yang et al. documented multiple foreign body granulomas developing shortly after the dermal injection of mesenchymal stem cell–conditioned media, illustrating a similar pattern of immune‐mediated reactions to injectable biologics [6]. Likewise, Nahm et al. and others have reported persistent erythematous, indurated papules and nodules following aesthetic exosome treatment, further underscoring the reproducibility of these complications when unregulated formulations are used [7].

Several immunopathological mechanisms may underlie the cutaneous adverse reactions observed following the intradermal injection of exosome‐containing formulations. These include delayed‐type hypersensitivity responses to xenogeneic or plant‐derived components [6, 7], and chronic granulomatous inflammation resulting from poorly degradable excipients or bio‐incompatible constituents [5, 8]. Moreover, innate immune activation may be triggered by damage‐associated molecular patterns or residual cellular debris present in unrefined or inadequately characterized exosome preparations, particularly when not manufactured under pharmaceutical‐grade conditions [8]. Immunogenicity related to the exosome source (e.g., mesenchymal stem cells or conditioned media) and the presence of bacterial byproducts, endotoxins, or undefined stabilizers may contribute to prolonged cutaneous inflammation and immune dysregulation in the dermis [4, 8].

These findings highlight the importance of using only medically approved injectable products within their intended indications, and ensuring that healthcare professionals administering these treatments have proper training in both technique and managing complications. Public education is equally vital, as many patients remain unaware of the risks associated with off‐label cosmetic injections. Without stronger regulatory oversight, standardized guidelines, and further research, the incidence of inflammatory reactions, persistent nodules, and scarring is likely to continue to increase; this poses a significant challenge in dermatological and aesthetic practice.

This case series has some limitations. First, histopathological confirmation was not performed, which precluded a definitive diagnosis of granulomatous or other specific inflammatory reactions. Second, treatment was incomplete. Since the injections were performed in non‐medical settings, subsequent therapies were limited to corticosteroids, lasers, or simple removal procedures. Other systemic anti‐inflammatory agents, such as methotrexate and cyclosporine, have not been investigated. This limitation reduces the therapeutic insights obtained in the present study. Finally, this was a small retrospective case series and the findings cannot be generalized to all patients receiving exosome‐based formulations. Larger controlled studies with standardized products and protocols are required to clarify the true safety profiles of these treatments.

## 4 Conclusion

This case series highlights the risks associated with the unauthorized intradermal injection of exosome‐containing formulations. Although approved for topical use, injecting these formulations can cause severe inflammation, persistent nodules, and scarring. Preventing such complications requires stronger regulatory enforcement to ensure products are used only as intended. Additionally, healthcare providers need proper education to follow evidence‐based practices and mitigate potential risks. Finally, raising public awareness about the dangers of off‐label cosmetic injections is crucial. Together, these measures can help minimize adverse events, protect patient safety, and uphold dermatological standards of care.

## Author Contributions

Kui Young Park was solely responsible for the conception and design of the study, data collection, analysis and interpretation, drafting of the manuscript, and critical revision of the content.

## Conflicts of Interest

The author declares no conflicts of interest.

## Data Availability

The datasets used and/or analyzed during the current study are available from the corresponding author on reasonable request.
